# Perceived Transparency from Dynamic Luminance Modulation in Uniform Center–Surround Displays

**DOI:** 10.3390/vision10010008

**Published:** 2026-02-06

**Authors:** Soomin Kim, Sung-Ho Kim

**Affiliations:** Department of Psychology, Ewha Womans University, 52 Ewhayeodae-gil, Seodaemun-gu, Seoul 03760, Republic of Korea; soom2nkim@ewhain.net

**Keywords:** transparency perception, luminance contrast, center–surround display, contrast modulation

## Abstract

We report a novel phenomenon in which dynamic changes in luminance are perceived as changes in transparency rather than as changes in surface lightness. Participants viewed an achromatic disc on a uniform gray background and indicated whether the observed change was best described in terms of lightness or transparency. In Experiment 1, transparency-change responses were more frequent at low contrast and were strongly biased toward sequences in which contrast decreased over time, revealing a pronounced asymmetry between decreasing and increasing contrast trajectories. Experiment 2 introduced a size manipulation, such that the disc either expanded or contracted during the luminance modulation. Transparency-change responses were highest when contrast decreased and the disc expanded, indicating that spatial expansion further amplifies transparency-related interpretations of the disc’s surface appearance. Overall, the results reveal a systematic asymmetry in how contrast-change direction shapes visual appearance, consistent with a forward bias in the processing of continuously changing visual signals. When contrast dynamically approached the background level, perceptual representations appeared to be weighted toward the upcoming low-contrast state, enhancing impressions of increasing transparency. These findings demonstrate that even minimal displays lacking traditional geometric cues to transparency can evoke strong transparency impressions, driven by predictive weighting of spatiotemporal contrast trajectories rather than by static image properties alone.

## 1. Introduction

We report a novel phenomenon in which luminance change is preferentially perceived as change in transparency (or opacity). While demonstrating a simple animation in a Matlab programming class—featuring a disc whose luminance cycled back and forth between black and white on a uniform mid-gray background—the second author noticed an unusual perceptual impression. Rather than appearing to merely change in lightness, the disc seemed to undergo a cyclic change in material appearance, alternating between more opaque- and more transparent-looking states, despite the absence of explicit transparency cues (see Video S1).

Importantly, this transparency-related experience was not symmetric over the luminance cycle. As the disc’s luminance approached that of the background—whether from higher luminance (white toward gray) or from lower luminance (black toward gray)—its contrast appeared to diminish rapidly, giving rise to an early and pronounced impression of increasing transparency. By contrast, once the disc crossed the background luminance and moved away from it—either toward higher luminance (gray toward white) or toward lower luminance (gray toward black)—perceived contrast appeared to increase abruptly, and the disc quickly regained a solid, opaque appearance. Thus, although the physical luminance modulation was continuous and approximately linear, the resulting perceptual experience was markedly nonlinear and asymmetric, with contrast reductions toward the background exerting a disproportionately strong influence on perceived transparency.

In computer graphics, luminance changes within a uniform region on a uniform background can be visually equivalent to changes in transmittance (i.e., the alpha channel), rendering such displays formally ambiguous. Accordingly, a homogeneous center–surround display (e.g., the individual frames) does not preclude an interpretation of a transparent disc over a uniform background. Crucially, however, this interpretation is not readily available perceptually and typically does not arise spontaneously in static viewing conditions.

According to traditional theories of perceptual transparency, e.g., [1,2], the perception of transparency requires at least four distinct image regions arranged in a way that forms X-junctions—points where contours intersect to imply overlapping surfaces (for illustrative examples). On this account, perceptual layering is thought to arise from such geometric configurations, and a homogeneous field cannot produce the impression of transparency.

However, some studies have challenged these assumptions, demonstrating that even homogeneous center-surround displays can induce the impression of transparency, e.g., [3,4,5,6]. Masin and Idone [6] first noted that homogeneous center-surround configuration can elicit transparency perception under low contrast conditions. Although participants initially did not report transparency, they began to perceive it—especially at low contrast—once the concept of transparency was introduced through instructions.

Building on these insights, Ekroll and colleagues [4,5] proposed that the perception of transparency in such simple displays may underlie simultaneous color contrast effects. In their view, when a uniform disc on a uniform surround is perceived as a transparent layer, the visual system decomposes the observed color into two components: that of the transparent layer and that of the background. This layered interpretation, especially under low contrast, can produce systematic shifts in perceived color—effects typically attributed to contextual contrast mechanisms. Schmid and Anderson [7] similarly showed that transparency perception may contribute to the perception of lightness in homogeneous center-surround stimuli. Allowing observers to adjust both lightness and transmittance led to more satisfactory matches, suggesting that the visual system may infer a transparent layer to reconcile edge contrast with perceived surface properties.

Motivated by the serendipitous observation, the present study examined whether dynamic modulation of luminance contrast—extending previous findings from static displays—can induce the perception of transparency. Specifically, we asked whether observers tend to interpret gradual luminance changes in a target as changes in transparency rather than in lightness. By focusing on spatiotemporal contrast trajectories rather than static snapshots, the present study investigated whether perceptual interpretations arising from temporal structure differ from those that could be inferred from comparisons between static images alone. Under a purely static comparison account, the direction of contrast change should not matter, as the same pair of images would be available regardless of temporal order. Any systematic effect of contrast-change direction would therefore point to a genuinely dynamic contribution to perceptual interpretation.

More broadly, this question connects to the possibility that continuously evolving visual signals engage predictive or forward-weighted perceptual mechanisms. Prior work on dynamic perception suggests that smoothly changing stimuli can be represented with a bias toward upcoming states of the signal [8,9]. The asymmetries observed in the present demonstration raise the possibility that transparency perception may be shaped in part by such forward weighting of spatiotemporal contrast, an issue we return to in the General Discussion.

To minimize potential confounds in observers’ judgments and to systematically examine the effects of contrast level and contrast-change direction, we employed simplified stimuli in which luminance modulation occurred in a single direction (either increasing or decreasing contrast) and was restricted so that the disc’s luminance did not cross the background level. Luminance changes in a target that cross the background level can produce qualitative changes in appearance (e.g., the apparent disappearance and reappearance of the disc), thereby complicating perceptual interpretation. Moreover, stimuli that become indistinguishable from the background may be especially prone to being interpreted as fully transparent. From this perspective, the present stimulus design represents a conservative choice, aimed at reducing response compounding and avoiding an overestimation of transparency-related judgments.

We addressed three primary questions regarding the conditions under which transparency perception emerges. First, would transparency-change responses occur more frequently when the luminance contrast between the target and background is low? Based on prior research [5,6], we predicted that lower contrast would yield stronger impressions of transparency. Second, we asked whether the direction of contrast change would influence perception. Specifically, would a luminance change toward the background level (i.e., decreasing contrast) produce stronger transparency impressions than a change away from it (i.e., increasing contrast)? If low contrast is critical, transparency might be triggered early during the change and persist throughout due to perceptual hysteresis, potentially leading to asymmetries in how the same contrast range is interpreted depending on the direction of change. Third, we examined whether transparency-change responses would be further amplified when contrast change was accompanied by spatial expansion. We hypothesized that this combination would bias observers toward interpreting the target’s surface appearance in transparency-like terms, thereby increasing the likelihood of transparency-change reports. This hypothesis was motivated by a phenomenological regularity in visual experience, whereby many semi-transparent materials—such as translucent membranes—appear more transparent when they expand or spread.

## 2. Experiment 1

### 2.1. Method

#### 2.1.1. Participants

Thirty Korean participants (16 females, aged 18 to 60 years) were recruited through online community advertisements at Ewha Womans University. They completed a 15-min online session in exchange for a small monetary payment. This study was approved by the university’s institutional review board. Written informed consent was waived on the condition that the experiment was conducted online. The sample size (*n* = 30) was determined a priori based on power analysis using G*Power 3.1.9.7 [10], which indicated that 24 participants would be required to achieve at least 80% power (α = 0.05) to detect a medium effect size (Cohen’s f = 0.25).

#### 2.1.2. Stimuli

The experiment was controlled by a script written in PsychoPy (ver. 2021.1) [11] and administered online via Pavlovia (http://pavlovia.org). As participants used their own personal computers, hardware specifications (e.g., screen resolution, monitor size, viewing distance, and display calibration) could not be controlled. Accordingly, the exact luminance output and its linearity could not be guaranteed across displays. References to linear luminance modulation in the manuscript therefore denote the linear interpolation of RGB values over time, rather than strictly linear changes in physical luminance.

**Figure 1:**
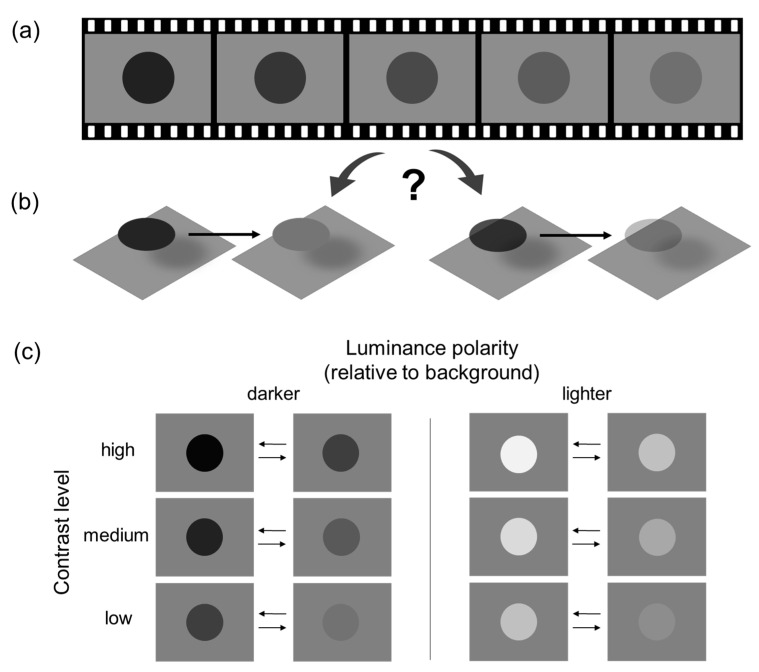
(**a**) Schematic depiction (not to scale) of the animation sequence used in Experiment 1. (**b**) Two competing interpretations of the dynamic display in (**a**): a change in lightness (**left**) versus a change in transparency (**right**). (**c**) Stimulus conditions used in Experiment 1. The luminance of the circular target either increased or decreased over time, resulting in either increasing or decreasing luminance contrast relative to the background. The left panel shows conditions where the target was darker than the background, and the right panel shows conditions where it was lighter. The three rows correspond to three levels of luminance contrast (high, medium, and low). In each cell, the pair of horizontal arrows indicates the direction of luminance change: either toward the background level (rightward arrow), resulting in decreasing contrast, or away from the background level (leftward arrow), resulting in increasing contrast. Animated demos can be viewed in Video S2.

Figure 1a shows a schematic illustration of the stimulus display (animated demos can be viewed in Video S2). On each trial, an achromatic circular target (diameter = 30% of the screen height) appeared at the center of a mid-grey background (PsychoPy RGB [0, 0, 0]) for 200 ms. The disc’s RGB values were linearly interpolated over 1800 ms, resulting in a gradual change in displayed luminance, either increasing or decreasing, without crossing the mid-gray level—thus maintaining its luminance polarity relative to the background throughout the change.

More specifically, the magnitude of RGB intensity change was fixed at 0.4 in PsychoPy RGB units (range: −1 to 1), defined as the normalized deviation of the stimulus RGB value from mid-gray (RGB = 0). Based on the absolute RGB intensity difference between the target and the background, three overlapping contrast levels were defined (Figure 1c): low (±0.1 to ±0.5), medium (±0.3 to ±0.7), and high (±0.5 to ±0.9). For each level, luminance varied between two fixed endpoints in one of two directions: either toward the background (decreasing contrast) or away from it (increasing contrast). For example, a target could darken from −0.5 to −0.9 (increasing contrast), or lighten from −0.9 to −0.5 (decreasing contrast; see Figure 1a).

#### 2.1.3. Procedure and Design

Participants completed the experiment on their own computers by following a link provided in the online advertisement. After giving informed consent, they were presented with instructions explaining how to distinguish two perceptual alternatives: changes in lightness versus changes in transparency.

**Figure 2:**
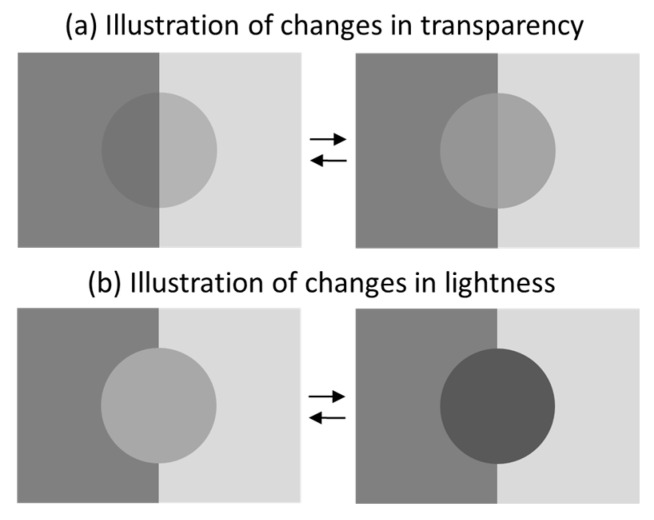
Instructional images used to illustrate perceived changes in transparency (**a**) and lightness (**b**), described to observers as follows: (**a**) A left-to-right change corresponds to a decrease in transparency (i.e., the disc becomes less transparent), whereas a right-to-left change corresponds to an increase in transparency (i.e., the disc becomes more transparent). (**b**) A left-to-right change corresponds to a decrease in lightness (i.e., the disc becomes darker), whereas a right-to-left change corresponds to an increase in lightness (i.e., the disc becomes lighter).

To illustrate a transparency change, participants viewed two side-by-side images of a bipartite rectangle (half light-grey, half dark-grey), each overlaid with a transparent-looking achromatic disc that appeared to differ in transmittance (i.e., prototypical four-region displays based on X-junctions). They were instructed that the difference between these discs could be interpreted as resulting from a change in transparency (for details, see Figure 2a). To illustrate a lightness change, participants then viewed another pair of bipartite rectangles, each containing a homogeneous grey disc that differed in lightness. They were told that the difference between these discs was intended to illustrate a change in lightness (for details, see Figure 2b).

Each trial began with a white fixation cross on a mid-grey background for 500 ms. A circular target then appeared for 200 ms, followed by a linear luminance change over 1800 ms. The target then disappeared, and participants pressed a key to indicate whether the change was perceived in terms of lightness or transparency.

The experiment consisted of five blocks of 24 trials each (120 trials in total). Each block included a full factorial combination of the following conditions: 2 luminance polarities (darker or lighter than the background) × 3 contrast levels (low, medium, high) × 2 contrast changes (increasing vs. decreasing) × 2 repetitions. Trial order was randomized within each block.

### 2.2. Results and Discussion

**Figure 3:**
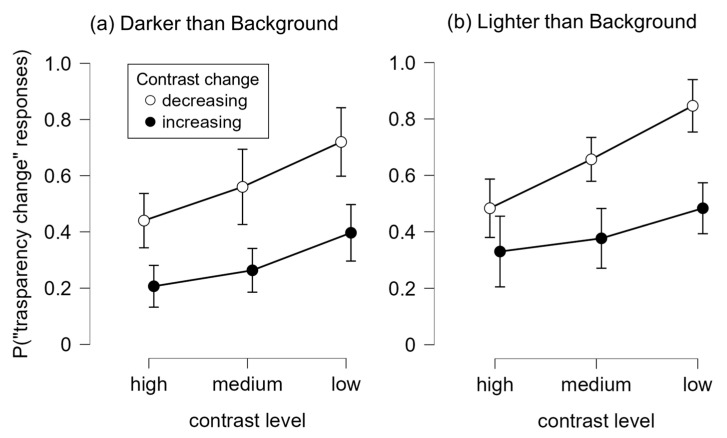
Mean proportion of transparency-change responses across conditions in Experiment 1, shown separately for darker-than-background targets (**a**) and lighter-than-background targets (**b**). Open circles indicate contrast-decreasing trials, and filled circles indicate contrast-increasing trials. Error bars represent 95% confidence intervals.

Figure 3 depicts the results of Experiment 1. A 3 × 2 × 2 repeated-measures analysis of variance (ANOVA) was conducted with contrast level (low, medium, high), contrast change (increasing vs. decreasing), and luminance polarity (darker vs. lighter than the background) as within-subject factors. Mauchly’s test indicated violations of sphericity for the contrast level factor, so Greenhouse–Geisser corrections were applied.

There was a significant main effect of contrast level, *F*(1.21, 35.07) = 25.95, *p* < 0.001, *η_p_*^2^ = 0.47, such that transparency-change responses increased as contrast level decreased—from high (*M* = 0.37, *SE* = 0.03) to medium (*M* = 0.46, *SE* = 0.02) to low (*M* = 0.61, *SE* = 0.03). A significant main effect of contrast change was also observed, *F*(1, 29) = 26.02, *p* < 0.001, *η_p_*^2^ = 0.47; participants were more likely to report a change in transparency when contrast decreased (*M* = 0.62, *SE* = 0.03) than when it increased (*M* = 0.34, *SE* = 0.04). The main effect of luminance polarity was not significant, *F*(1, 29) = 3.82, *p* = 0.060, ηp^2^ = 0.12, despite a numerical trend toward higher transparency responses in the lighter-than-background condition (darker-than-background: *M* = 0.431, *SE* = 0.03; lighter-than-background: *M* = 0.529, *SE* = 0.04).

A significant interaction emerged between contrast level and contrast change, *F*(2, 57.95) = 7.45, *p* = 0.001, *η_p_*^2^ = 0.20, indicating that the influence of contrast level was more pronounced when contrast was decreasing. Simple main effect analyses revealed a significant effect of contrast level in both contrast change conditions (*ps* < 0.01). However, the increase in transparency-change responses from high to low contrast was significantly greater in the decreasing contrast condition (*M*_difference_ = 0.32) than in the increasing condition (*M*_difference_ = 0.17), *t*(29) = 3.79, *p* < 0.001, *d* = 0.69. This pattern suggests that contrast reduction not only enhances the likelihood of transparency perception overall, but also amplifies the visual system’s sensitivity to variations in contrast magnitude. All other two-way and three-way interactions were not significant (*ps* > 0.32).

The results of Experiment 1 support the idea that transparency perception is closely tied to luminance contrast. Transparency-change responses were more frequent when the contrast between the target and background was low, confirming that reduced contrast facilitates a transparency interpretation. This finding is consistent with prior work using static displays [5,6] and extends the role of low contrast to dynamic luminance changes.

Critically, the direction of contrast change influenced perception: Transparency-change responses were more frequent when contrast decreased (i.e., as the target approached the background luminance) than when it increased. This asymmetry runs counter to a simple perceptual hysteresis account, which would predict that once a transparency interpretation is triggered at low contrast, it should persist during subsequent increases in contrast. Rather than reflecting such persistence, the results reveal a systematic bias favoring transparency-change reports during decreasing-contrast trajectories.

At this stage, the present data do not allow us to adjudicate between alternative explanations of this asymmetry. One possibility is that observers’ judgments are disproportionately influenced by the terminal phase of the sequence, such that decreasing-contrast trajectories—ending at low contrast—are more likely to elicit transparency-change responses. Another possibility is that the visual system exhibits a forward bias when processing continuously changing stimuli, such that perception is weighted toward the upcoming state of a smoothly evolving contrast signal. Similar forward biases have been documented for continuously changing features such as luminance and color, where a dynamic stimulus is perceived as leading a briefly flashed reference in feature space [9]. These possibilities are examined further in the context of Experiment 2 and in the General Discussion.

## 3. Experiment 2

In everyday visual experience, semi-transparent surfaces often appear more transparent when they are extended over a larger area (e.g., the surface of an inflated balloon). Such statistical regularities may bias the visual system toward interpreting changes in an object’s size in transparency-like terms, even when no material change is present.

Building on the finding that dynamic luminance changes bias appearance toward transparency, Experiment 2 examined whether spatial expansion further enhances this effect. The target either expanded or contracted in synchrony with its luminance modulation, allowing us to test whether this spatial cue selectively modulates transparency judgments, particularly under decreasing-contrast trajectories.

### 3.1. Method

**Figure 4:**
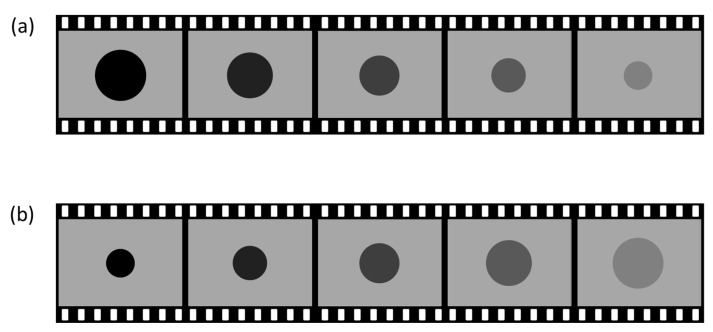
Both are the sequence of stimuli used in Experiment 2. (**a**) represents that the contrast between the circle and the background decreases while contracting the size. (**b**) represents that the contrast decreases while expanding the size.

Experiment 2 was identical to Experiment 1 except for the following changes. Thirty new observers (28 females; age range: 19–50 years) participated. The circular target either contracted (diameter decreased from 40% to 15% of screen height) or expanded (15% to 40%) in synchrony with its luminance modulation (Figure 4; see also Video S3 for animated demos).

Because size change was introduced as a new within-participant factor, luminance polarity—manipulated within participants in Experiment 1—was varied between participants, given its non-significant effects previously. Thus, Experiment 2 followed a mixed factorial design with one between-participant factor (luminance polarity: target darker or lighter than the background) and three within-participant factors: contrast level, contrast-change direction, and size change. Participants completed five blocks of 24 trials (2 repetitions × 12 within-participant conditions), with trial order randomized within each block.

### 3.2. Results and Discussion

An initial four-way mixed-design ANOVA revealed no significant main effect or interaction involving luminance polarity. Therefore, data were collapsed across this between-participant factor and analyzed using a 2 × 3 × 2 (contrast change direction × contrast level × size change) repeated-measures ANOVA.

**Figure 5:**
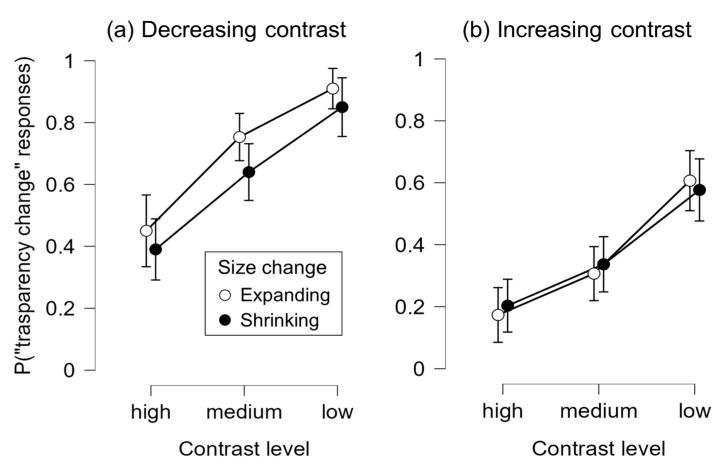
Mean proportion of the transparency perception across the conditions in Experiment 2. Error bars represent 95% CIs.

Figure 5 depicts the results of Experiment 2. The analysis revealed a significant main effect of contrast-change direction, *F*(1, 29) = 36.06, *p* < 0.001, *η_p_*^2^ = 0.55, with more transparency-change responses with decreasing contrast (*M* = 0.67, *SE* = 0.04) than with increasing contrast (*M* = 0.37, *SE* = 0.05). A main effect of contrast level was also significant, *F*(2, 58) = 48.83, *p* < 0.001, *η_p_*^2^ = 0.63, with transparency-change responses increasing as contrast decreased: from high (*M* = 0.30, *SE* = 0.05) to medium (*M* = 0.51, *SE* = 0.04) to low (*M* = 0.74, *SE* = 0.04) contrast levels. The main effect of size-change direction was not significant, *F*(1, 29) = 3.13, *p* = 0.088, *η_p_*^2^ = 0.10.

As in Experiment 1, there was a significant interaction between contrast-change direction and contrast level, *F*(2, 58) = 5.49, *p* = 0.007, *η_p_*^2^ = 0.16, even though post hoc comparisons showed that all adjacent contrast levels differed significantly in both contrast-change conditions, *ps* < 0.05. Critically, a significant interaction emerged between contrast-change direction and size-change direction, *F*(1, 29) = 8.39, *p* = 0.007, *η_p_*^2^ = 0.22. When contrast decreased, expanding targets elicited significantly more transparency-change responses (*M* = 0.70, *SE* = 0.02) than contracting targets (*M* = 0.63, *SE* = 0.02), *F*(1, 29) = 8.21, *p* = 0.008. No such difference was observed when contrast increased (*M*s = 0.36 vs. 0.37, *SE* = 0.02), *F*(1, 29) = 0.22, *p* = 0.644.

Experiment 2 replicated the key findings from Experiment 1: transparency-change responses increased with decreasing contrast magnitude, and were particularly frequent when contrast decreased over time. These results confirm that transparency perception is modulated by both the magnitude of contrast and its temporal trajectory.

Importantly, the interaction between contrast change and size change revealed an additional modulation. Spatial expansion enhanced transparency perception, but only in the context of decreasing contrast; when contrast increased, size change had no effect. This pattern suggests that spatial expansion selectively strengthens transparency impressions under conditions in which dynamic contrast reduction already biases appearance toward transparency.

Together, these findings show that perceived transparency depends not only on the magnitude of contrast and the direction of contrast change, but also on spatiotemporal cues of surface expansion that are phenomenologically associated with increased transparency. In particular, the combination of decreasing contrast and expansion appears to function as a perceptual heuristic that biases observers toward transparency-based interpretations of dynamic changes in surface appearance, even in the absence of explicit geometric cues to transparency.

## 4. General Discussion

Across two experiments, we investigated a novel phenomenon in which gradual luminance modulation of a uniform disc on a uniform background was perceived not simply as a change in lightness, but as a change in transparency. In both experiments, transparency-change responses were more likely when the luminance contrast between the disc and background was low, and especially when contrast decreased over time. Experiment 2 extended this finding by showing that contrast reduction paired with spatial expansion further enhanced the perception of transparency change. Together, these findings demonstrate that even in minimal displays lacking traditional geometric cues to transparency, such as X-junctions, dynamic luminance and spatial changes can elicit strong impressions of transparency.

The increased transparency-change responses under low-contrast conditions are consistent with previous findings using static center-surround displays, e.g., [6], which showed that even minimal two-area configuration can produce transparency impressions when contrast is low. The present results extend these findings by showing that such impressions are not only supported by static contrast relations, but are further strengthened by dynamic contrast modulation.

This pattern is also consistent with Ekroll and Faul’s proposal [4] that simultaneous contrast effects—and possibly transparency itself—result from an implicit decomposition of a visual input into a transparent layer and background. In the present study, the low—and especially decreasing—contrast reliably biased observers toward transparency-based interpretations, with such responses occurring in a substantial majority of trials (over 80%). Importantly, these results indicate that transparency perception is not determined solely by instantaneous contrast levels, but is dynamically shaped by how contrast evolves over time.

One possible interpretation of this finding is that the integration of the changing luminance contrast between the center and surround over time would create a spatiotemporal structure that functions analogously to an X-junction. These temporal luminance transitions may promote perceptual scission by suggesting the emergence or dissipation of a transparent layer. This idea resonates with previous suggestions that dynamic scene changes can substitute for static occlusion cues like T-junctions in surface interpretation [12].

The direction of contrast change played a critical role, revealing a consistent perceptual asymmetry across both experiments: Decreasing contrast was more effective in promoting perceptual scission into a transparent layer and background than increasing contrast, even when overall contrast levels were comparable. This asymmetry challenges the idea that transparency, once triggered, would simply persist due to perceptual hysteresis. Instead, the results suggest that the visual system is more attuned to the emergence than to the disappearance of transparency, favoring interpretations where increasing similarity between target and background signals a change in the target’s surface transparency.

Beyond contrast dynamics, Experiment 2 further demonstrated that transparency perception is modulated by spatial expansion. When contrast decreased, expanding targets elicited more frequent transparency-change responses than contracting ones, indicating that radial expansion enhances transparency-related interpretations of the target’s surface appearance. This pattern suggests that the visual system integrates multiple dynamic cues—such as decreasing contrast and spatial expansion—as heuristics for interpreting surface structure.

This interpretation is ecologically grounded: In everyday visual experience, objects or surfaces often appear less dense and more transmissive when they expand or spread over a larger area, as in the case of stretching thin films or inflating translucent materials. Accordingly, changes in size and luminance may become perceptually linked through learned associations with physical processes, biasing observers toward transparency-based interpretations.

One reviewer raised the possibility that radial expansion of the disc might function as a cue to depth or looming. Because size modulation in the present study was gradual and linear rather than abrupt or exponential, we presume that observers could experience a weak or implicit impression of approach. This possibility is not incompatible with our interpretation. In everyday experience, semi-transparent surfaces—such as thin membranes or films—often appear more transparent when they expand or are viewed at closer distances. Accordingly, an impression of approach could further amplify the transparency-related interpretations already associated with the disc’s expansion.

One might also ask whether the dynamic impressions observed here could be related to a dynamic instantiation of the crispening effect, which refers to enhanced lightness discriminability near the background luminance [7,13,14]. However, the crispening effect primarily concerns static differences in lightness discriminability as a function of luminance level and does not make specific predictions about directional asymmetries in contrast change over time. In contrast, the present results reveal a robust asymmetry in transparency perception depending on the direction of contrast change: transparency-related responses were consistently more prevalent during contrast decreases than increases, even when overall contrast levels were comparable. This dissociation suggests that the observed effects reflect a distinct dynamic perceptual mechanism rather than a dynamic counterpart of the crispening effect.

In a sense, this asymmetry suggests that transparency perception is influenced more by the final than by the initial state of a dynamic sequence. A straightforward account of this asymmetry is a recency-based or end-state bias. Because decreasing-contrast sequences necessarily terminate at low contrast and increasing-contrast sequences at high contrast, observers’ judgments may have been disproportionately influenced by the final contrast level of the event.

A strict end-state account therefore predicts that conditions sharing the same final contrast level—namely, increasing contrast from a low level and decreasing contrast from a high level—should elicit comparable transparency-change responses. This prediction was supported in Experiment 1, where no reliable difference was observed between these two conditions (*t*(29) = 0.41, *p* = 0.685, *d* = 0.08). In Experiment 2, however, transparency-change responses were significantly more frequent in the low-contrast increasing condition than in the high-contrast decreasing condition, despite identical end states (*t*(29) = 2.18, *p* = 0.037, *d* = 0.70). Taken together, these results indicate that transparency judgments were not determined solely by the terminal contrast level.

Along this line of thought, the present findings point to a role for global properties of the luminance trajectory—rather than the end state alone—and, more specifically, to a forward bias in the processing of continuously changing visual signals. When a stimulus changes smoothly over time, perceptual representations may be weighted toward the upcoming state of the signal, rather than reflecting only its instantaneous value, a phenomenon commonly referred to as the flash-lag effect [8]. Similar forward biases have been documented for continuously changing non-spatial features such as luminance and color, where a dynamic stimulus is perceived as leading a briefly flashed reference in feature space [9].

The phenomenological asymmetry observed in the demonstration that motivated the present study, together with the results of both experiments, suggests a close link between transparency perception and such forward bias mechanisms. When luminance contrast dynamically approached the background level, a forward bias toward the upcoming low-contrast state may have exaggerated the perceived rate of contrast reduction, thereby causing the stimulus to enter a low-contrast regime earlier and more strongly—a condition known to promote transparency interpretations. Conversely, when contrast moved away from the background, the same forward bias would favor the upcoming high-contrast state, causing the stimulus to more rapidly enter a contrast regime associated with opaque surface appearance. From this perspective, the observed asymmetry does not reflect a simple sensitivity to low contrast per se, but rather the operation of a predictive or extrapolative mechanism that biases the evolution of perceived contrast, which in turn modulates the likelihood of transparency-based interpretations.

Along this line of argument, the present findings can also be related to representational momentum–like effects [15], which reflect a general tendency to weight the future state of a changing signal. Although representational momentum has typically been studied in memory tasks involving implied motion, it has also been shown to affect brightness judgments, such that brightening stimuli are remembered as brighter and darkening stimuli as darker than they actually were [16]. If such a bias extends to dynamic changes in center–surround contrast, it could further exaggerate perceived contrast reduction over time, thereby increasing the likelihood of interpreting the display in transparency-related terms. Thus, representational momentum may contribute to transparency perception not only at the level of retrospective judgment, but also as part of an ongoing perceptual organization process.

As noted by one reviewer, decreasing-contrast sequences may give rise to an impression that the target becomes indistinguishable from the background toward the end of the sequence. This observation is consistent with the phenomenology that motivated the present study, in which the disc appeared to become transparent early as its luminance approached that of the background. We suggest that this experience is more plausibly attributed to a forward bias or momentum-like extrapolation process—whereby the visual system extrapolates the ongoing contrast reduction beyond the physically presented stimulus range—than to an actual match between target and background luminance.

Another alternative interpretation is that the observed transparency bias reflects a positive afterimage or boundary persistence, rather than dynamic perceptual organization. On this view, boundary-related signals might briefly persist and become dissociated from surface-related signals, giving rise to a transient impression of transparency. However, several aspects of the present design argue against this account. The stimulus duration (1800 ms) is short for producing robust afterimage effects, any positive persistence is typically brief, and stimulus offset was immediately followed by a response screen likely to act as a visual mask. Moreover, in Experiment 2 the disc boundary changed continuously over time, making the formation of a stable boundary afterimage unlikely. Critically, if boundary persistence were the primary driver, similar transparency biases should be observed for both increasing- and decreasing-contrast trajectories, which was not the case.

A related low-level explanation concerns visual adaptation. Classic adaptation effects typically require prolonged exposure to stable stimulus properties and are often maintained by top-up adaptation. By contrast, the present stimuli involved brief, continuously changing luminance trajectories, with different contrast directions interleaved across trials. Under these conditions, there was no stable stimulus level to which the visual system could plausibly adapt. Taken together, these considerations suggest that neither afterimage-based nor conventional adaptation accounts can adequately explain the observed asymmetries, which are more consistent with a dynamic, forward-biased interpretation of evolving contrast trajectories.

An important question raised by the present findings concerns whether the observed asymmetries truly result from visual processing of continuous dynamic modulation, or whether they could instead reflect post-perceptual reasoning based on static information or task demands [17]. In principle, if the present effects were driven primarily by higher-level judgment, they should be reproducible by presenting observers with only static snapshots corresponding to the beginning and end of a contrast sequence (as illustrated in Figure 1c), allowing them to infer that a change has occurred.

A related concern is that the observed transparency bias may partly reflect demand characteristics. Because observers were explicitly asked to categorize the change in appearance as either a change in lightness or a change in transparency, the task structure may have encouraged conceptual or judgment-based strategies. In particular, when one response option is perceived as more obvious or conventional, observers may be inclined to select the alternative option, potentially inflating transparency-change reports.

However, these judgment-based accounts face a critical limitation. Transparency perception is often ambiguous in static low-contrast displays and may require instruction or conceptual framing to be reported reliably. Even if observers were able to interpret the static endpoints in transparency-related terms—perceiving the low-contrast image as transparent and the high-contrast image as opaque—it remains unclear how they would interpolate the intermediate perceptual states. A simple interpolation between “transparent” and “opaque” endpoints would not naturally predict the pronounced asymmetries observed here with respect to contrast direction and rate of change. From this perspective, the present findings suggest that dynamic contrast trajectories play a substantive role in shaping transparency perception, beyond what can be inferred from static contrast relations or task demands alone.

In sum, our findings show that even minimal displays lacking traditional structural cues can elicit strong impressions of transparency when guided by appropriate spatiotemporal dynamics. Importantly, transparency perception was shaped not only by instantaneous contrast relations, but by the temporal trajectory of contrast change, with decreasing contrast exerting a disproportionate influence on appearance. These results suggest that the visual system does not merely register static image properties, but actively interprets evolving signals by weighting their future states, consistent with forward bias mechanisms previously documented for dynamic visual features. More broadly, the present findings contribute to a growing understanding of how surface structure is flexibly inferred from dynamic image information.

## Data Availability

The data that support the findings of this study are available from the corresponding author, upon request.

## Supplementary Materials

The following supporting information can be downloaded at: https://www.mdpi.com/article/10.3390/vision10010008/s1, Video S1: Animated demonstration of the display that motivated the present study (A uniform disc is presented on a mid-gray background while its displayed luminance is modulated cyclically over time). Video S2: Animated demonstrations of the stimulus displays used in Experiment 1. Video S3: Animated demonstrations of the stimulus displays used in Experiment 2.
